# Negative association between serum calcium and glucocorticoid-induced hypertension in thyroid-associated ophthalmopathy patients treated with methylprednisolone

**DOI:** 10.3389/fendo.2025.1548953

**Published:** 2025-04-11

**Authors:** Ling Wang, Yushi Sun, Meng Zhang, Hairong He, Jingya Wang, Huayang Xu, Yang Hao, Wenqiang Zhang, Yawen Wang, Kelvin K. L. Chong, Hui Guo, Bingyin Shi, Yue Wang

**Affiliations:** ^1^ Department of Endocrinology, The First Affiliated Hospital of Xi’an Jiaotong University, Xi’an, China; ^2^ Center of Investigator Initiated Trials, The First Affiliated Hospital of Xi’an Jiaotong University, Xi’an, China; ^3^ Department of Gastroenterology, Xi’an Children’s Hospital, Shaanxi Research Institute for Pediatric Diseases, The Affiliated Children’s Hospital of Xi’an Jiaotong University and National Regional Medical Center for Children (Northwest), Xi’an, China; ^4^ Department of Ophthalmology, The First Affiliated Hospital of Xi’an Jiaotong University, Xi’an, China; ^5^ Department of Laboratory Medicine, The First Affiliated Hospital of Xi’an Jiaotong University, Xi’an, Shaanxi, China; ^6^ Biobank, The First Affiliated Hospital of Xi’an Jiaotong University, Xi’an, Shaanxi, China; ^7^ Shaanxi Engineering Research Center for Biobank and Advanced Medical Research, The First Affiliated Hospital of Xi’an Jiaotong University, Xi’an, Shaanxi, China; ^8^ Department of Ophthalmology and Visual Sciences, The Prince of Wales Hospital, Hong Kong, Hong Kong SAR, China; ^9^ Department of Ophthalmology and Visual Sciences, Faculty of Medicine, The Chinese University of Hong Kong, Hong Kong, Hong Kong SAR, China; ^10^ Hong Kong Eye Hospital, Hong Kong, Hong Kong SAR, China

**Keywords:** serum calcium, hypocalcemia, hypertension, glucocorticoid, thyroid-associated ophthalmopathy

## Abstract

**Background:**

Hypertension is a common adverse event after systemic glucocorticoid therapy. Previous studies have suggested that blood pressure (BP) regulation is related to serum calcium. However, whether serum calcium affects the risk of glucocorticoid-induced hypertension remains understudied.

**Methods:**

We used data from thyroid-associated ophthalmopathy (TAO) patients who completed a course of intravenous methylprednisolone (IVMP). Patients with high BP at baseline, a history of hypertension, and missing data were excluded. Glucocorticoid-induced hypertension was defined as systolic BP (SBP) ≥ 140 mmHg or diastolic BP (DBP) ≥ 90 mmHg during follow-up. Multivariate logistic regression and generalized additive models were used to investigate the associations between serum calcium and glucocorticoid-induced hypertension. Bar charts were used to compare the SBP and DBP fluctuations between patients with and without hypocalcemia. After accounting for missing data, all analyses were repeated in the imputed cohort.

**Results:**

Serum calcium was negatively correlated with glucocorticoid-induced hypertension after adjusting for covariates with p-value < 0.1 (including age, body mass index, SBP, and DBP). For each 0.1 mmol/L increase in serum calcium, the OR (95% CI) was 0.61 (0.39, 0.95). Furthermore, a nonlinear relationship was observed, with an inflection point at 2.10 mmol/L. After the serum calcium level was converted into a categorical variable, hypocalcemia was positively associated with glucocorticoid-induced hypertension (OR = 3.26, 95% CI = 1.11–9.53). Patients with hypocalcemia exhibited significantly greater SBP fluctuations than patients without hypocalcemia (p < 0.05). These results were stable when adjusting for confounders and in the analyses of the imputed cohort.

**Conclusions:**

Hypocalcemia was associated with glucocorticoid-induced hypertension in TAO patients. Further research is needed to confirm these findings in larger populations and to investigate whether calcium supplementation before glucocorticoid therapy may reduce such risk.

## 1 Introduction

Graves’ disease (GD) is the most common organ-specific autoimmune disease, with an annual incidence of 20 to 50 cases per 100,000 people (1–3). Up to 50% of GD patients may develop thyroid-associated ophthalmopathy (TAO), which is characterized by eyelid swelling, erythema and retraction, conjunctival swelling, exophthalmos, and restrictive strabismus. In severe cases, intractable diplopia, vision loss, and even blindness may occur (1, 4, 5). TAO significantly affects the vision-related quality of life and general health of patients.

The treatment options for TAO are limited. Novel drugs such as teprotumumab, rituximab, tocilizumab, rapamycin, and azathioprine have been shown to be effective as monotherapies or combination therapies. Nevertheless, most drugs have not been approved by the FDA for use in TAO. In addition, there is controversy over the efficacy of rituximab in different studies. The efficacy of rapamycin and tocilizumab has only been confirmed in small sample clinical studies. Azathioprine has a slow onset of action. Although teprotumumab has been approved by the FDA, its cost is very high (6–14). Intravenous methylprednisolone (IVMP) is still the first-line treatment for moderate-to-severe and active TAO. The most common protocol employs a cumulative dose of 4.5 g methylprednisolone, given in 12 weekly infusions (six infusions of 0.5 g, followed by six infusions of 0.25 g) (4).

Long-term (3–6 months or longer) and high-dose (exceeding 30 mg/d) systemic glucocorticoids lead to many adverse consequences, particularly hypertension (15–18). According to a meta-analysis of randomized double-masked clinical trials, patients receiving systemic glucocorticoids have a 2.2 (1.4–3.8) times greater risk of developing hypertension than those who receive placebo (19). Furthermore, patients are at risk for cardio-cerebrovascular events even if their blood pressure (BP) is only momentarily increased (20). Patients may experience dizziness and headaches as a result. There are also reports that patients can experience a myocardial infarction due to a severe increase in blood pressure (21). Therefore, it is crucial to focus on the risk factors for glucocorticoid-induced hypertension.

Calcium is a rich mineral element in the human body and is involved in the contraction and relaxation of vascular smooth muscle cells, the regulation of serum lipid levels, and the release of multiple hormones. Its role in blood pressure control has received increasing attention. In a prospective cohort study, active serum calcium was shown to be protective against future hypertension in middle-aged males (22). Yao et al. reported a significantly lower BP in older men with increasing serum calcium (23). In addition, calcium supplements have been found to lower BP and prevent gestational hypertension and preeclampsia (24–26). Nevertheless, some contrary results have suggested that serum calcium is positively associated with the risk of hypertension (27, 28). However, glucocorticoid-induced hypertension is a type of secondary hypertension, and it is unclear whether the serum calcium level may have any effect on it.

We aimed to conduct a retrospective cohort study to investigate whether baseline serum calcium levels are associated with the risk of glucocorticoid-induced hypertension in TAO patients receiving IVMP therapy.

## 2 Methods

### 2.1 Study design and population

**Figure 1:**
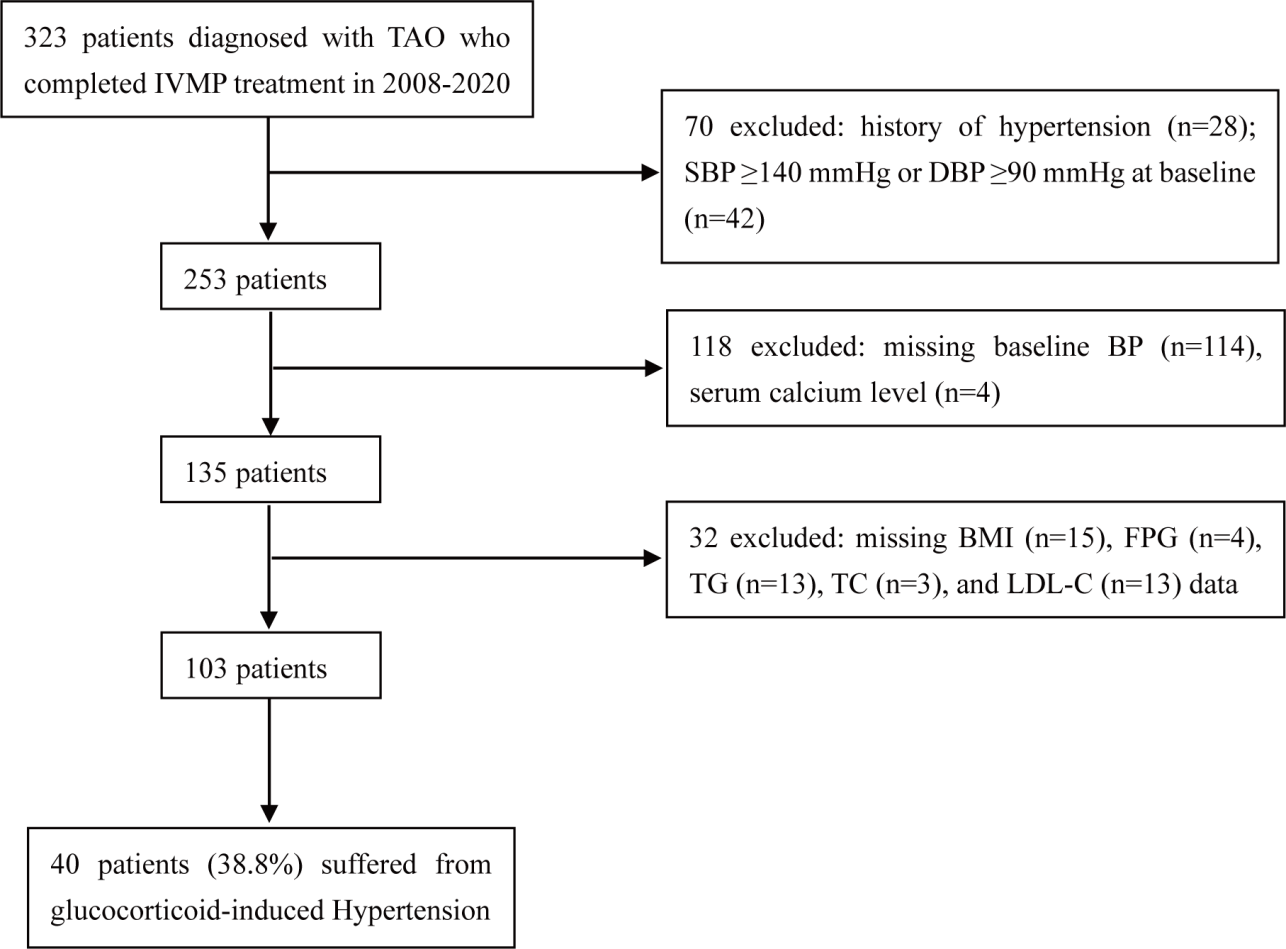
Study design and population. TAO, thyroid-associated ophthalmopathy; IVMP, intravenous methylprednisolone; SBP, systolic blood pressure; DBP, diastolic blood pressure; BP, blood pressure; BMI, body mass index; FPG, fasting plasma glucose; TG, triglyceride; TC, total cholesterol; LDL-C, low-density lipoprotein cholesterol.

We conducted a retrospective study of consecutive patients who were diagnosed with moderate-to-severe, active TAO and completed the full course of IVMP at the Department of Endocrinology, First Affiliated Hospital of Xi’an Jiaotong University, from January 2008 to July 2020. TAO was diagnosed on the basis of classic clinical manifestations and orbital CT scans. Patients with high BP at baseline, a documented history of hypertension, or missing data were excluded from this study. The exclusion criteria are shown in **Figure 1**. In total, 103 patients were enrolled in this study. This study was approved by the Ethics Committee of the First Affiliated Hospital of Xi’an Jiaotong University and was in line with the principles of the Helsinki Declaration on Clinical Research.

### 2.2 Data collection and outcome evaluation

The list of TAO patients receiving IVMP therapy was retrieved from the medical records system of the First Affiliated Hospital of Xi’an Jiaotong University. Baseline demographic data, smoking and drinking history, family history of hypertension, biochemical characteristics, and other relevant information were collected. These data were reviewed and cross-checked by three researchers to ensure their authenticity.

The regimen for IVMP treatment was 0.5–1.0 grams of methylprednisolone given every other day three times and was repeated at 3-week intervals for a total of 3 cycles. The choice of 500 mg or 1.0 g IVMP was determined by disease activity, severity, and the presence of any relative contraindications. The glucocorticoid dose was the total dose of methylprednisolone infused after the three treatment courses and ranged from 4.5 g to 9.0 g.

BP was measured with a mercury sphygmomanometer by trained nursing staff before and after each IVMP treatment and is expressed as the average value of two consecutive measurements. Glucocorticoid-induced hypertension was defined as systolic BP (SBP) ≥ 140 mmHg or diastolic BP (DBP) ≥ 90 mmHg during 3 visits to the hospital for glucocorticoid treatment.

Thyroid function was categorized as euthyroidism, hyperthyroidism, or hypothyroidism on the basis of serum free thyroid hormone (fT3, fT4) levels and sensitive thyroid stimulating hormone levels. The 7-item modified clinical activity score (CAS) based on the latest EUGOGO clinical practice guidelines includes eyelid redness, eyelid edema, conjunctival redness, conjunctival edema, caruncle or plica edema, spontaneous retrobulbar pain, and pain during eye movement (4).

### 2.3 Statistical analysis

Continuous variables are presented as the means ± SDs, and categorical variables are presented as the frequencies (percentages). The significance of each variable was assessed individually by univariate logistic regression analysis. Variables with p values less than 0.1 from the univariate analysis were considered significant covariates/confounders and were included in multivariate logistic regression analysis to estimate the independent association between the serum calcium level and glucocorticoid-induced hypertension. Bar–dot plots were used to display the data distribution of significant covariates/confounders in patients with and without glucocorticoid-induced hypertension. To further ensure the stability of the results, other variables were added one at a time into the multivariate logistic regression analysis.

In addition, generalized additive models were used to investigate any nonlinear associations between serum calcium and glucocorticoid-induced hypertension. Segmented regression and two-step recursion were used to identify the inflection point, which was then used to define the level of hypocalcemia. The relationship between hypocalcemia and glucocorticoid-induced hypertension was investigated using the multivariate logistic regression analysis, as previously mentioned. In addition, bar charts were used to compare the SBP and DBP fluctuations between patients with and without hypocalcemia.

### 2.4 Sensitivity analyses

To improve the statistical power and decrease bias that might have occurred if patients with missing data were excluded from the analyses, data imputation was performed on body mass index (BMI), fasting plasma glucose (FPG), triglyceride (TG), total cholesterol (TC), and low-density lipoprotein cholesterol (LDL-C) levels. When the proportion of missing data was less than 10%, the median was used to impute missing values. Otherwise, multiple imputation based on 5 replications and a chained equation approach method was used to impute missing values. We repeated all analyses with the imputed cohort (n = 135) for comparison.

All the statistical analyses were carried out using R software (version 4.2.0, http://www.R-project.org) and EmpowerStats (http://www.empowerstats.net).

## 3 Results

### 3.1 Baseline characteristics

After patients with missing data were excluded, 103 TAO patients were included in the complete cohort, 40 (38.8%) of whom suffered from glucocorticoid-induced hypertension (**Figure 1**). The baseline characteristics are listed in **Table 1**. Among these 103 TAO patients, 43.7% were female, the mean (SD) age was 47.6 (11.0) years, the mean CAS was 3.0, and 92.2% had hyperthyroidism before IVMP therapy. The mean SBP and DBP at baseline were 116.9 mmHg and 71.9 mmHg, respectively. A total of 12.6% of patients had a family history of hypertension. The mean serum calcium concentration was 2.20 mmol/L, which is within the lower limit of the normal range (2.20–2.60 mmol/L). In addition, 90.3% of patients were prescribed calcium supplements, and 63.1% of patients were prescribed vitamin D supplements to prevent or treat osteoporosis during treatment.

**Table 1:** Crude associations of glucocorticoid-induced hypertension with serum calcium and other potential risk factors in the complete cohort (n = 103).

Variable	Mean ± SD/n (%)	OR (95% CI)	P Value
Age, y	47.6 ± 11.0	1.04 (1.00, 1.08)	0.067
BMI, kg/m²	22.9 ± 3.0	1.21 (1.05, 1.40)	**0.009**
SBP, mmHg	116.9 ± 10.6	1.07 (1.02, 1.12)	**0.002**
DBP, mmHg	71.9 ± 7.8	1.09 (1.02, 1.15)	**0.006**
Serum calcium, mmol/L	2.20 ± 0.12	0.68 (0.46, 0.99)*	**0.046**
Serum albumin, g/L	41.1 ± 3.5	0.97 (0.87, 1.09)	0.614
Glucocorticoid dose, g	5.0 ± 1.3	1.12 (0.82, 1.52)	0.481
FPG, mmol/L	4.9 ± 1.3	0.90 (0.65, 1.26)	0.545
SCR, umol/L	55.0 ± 11.8	1.03 (0.99, 1.06)	0.545
TG, mmol/L	1.30 ± 0.75	1.50 (0.86, 2.62)	0.151
TC, mmol/L	4.02 ± 0.83	0.90 (0.55, 1.46)	0.658
LDL-C, mmol/L	2.39 ± 0.69	0.90 (0.50, 1.61)	0.720
CAS	3.0 ± 1.2	0.93 (0.66, 1.30)	0.656
Female	45 (43.7%)	0.92 (0.42, 2.06)	0.846
Smoking history	42 (40.8%)	1.12 (0.50, 2.51)	0.777
Drinking history	24 (23.3%)	1.17 (0.46, 2.96)	0.745
Family history of hypertension	13 (12.6%)	0.98 (0.30, 3.24)	0.976
Thyroid function:			
Euthyroidism	4 (3.9%)	Reference	
Hyperthyroidism	95 (92.2%)	0.64 (0.09, 4.73)	0.660
Hypothyroidism	4 (3.9%)	0.33 (0.02, 6.65)	0.472
Calcium supplements	93 (90.3%)	1.54 (0.37, 6.35)	0.549
Vitamin D supplements	65 (63.1%)	0.68 (0.30, 1.53)	0.348

*For each 0.1 mmol/L increase in serum calcium. Bold values indicate statistically significant results (p < 0.05).

BMI, body mass index; SBP, systolic blood pressure; DBP, diastolic blood pressure; FPG, fasting plasma glucose; SCR, serum creatinine; TG, triglyceride; TC, total cholesterol; LDL-C, low-density lipoprotein cholesterol; CAS, clinical activity score.

### 3.2 Univariate logistic regression analysis of glucocorticoid-induced hypertension with serum calcium and other potential risk factors

**Figure 2:**
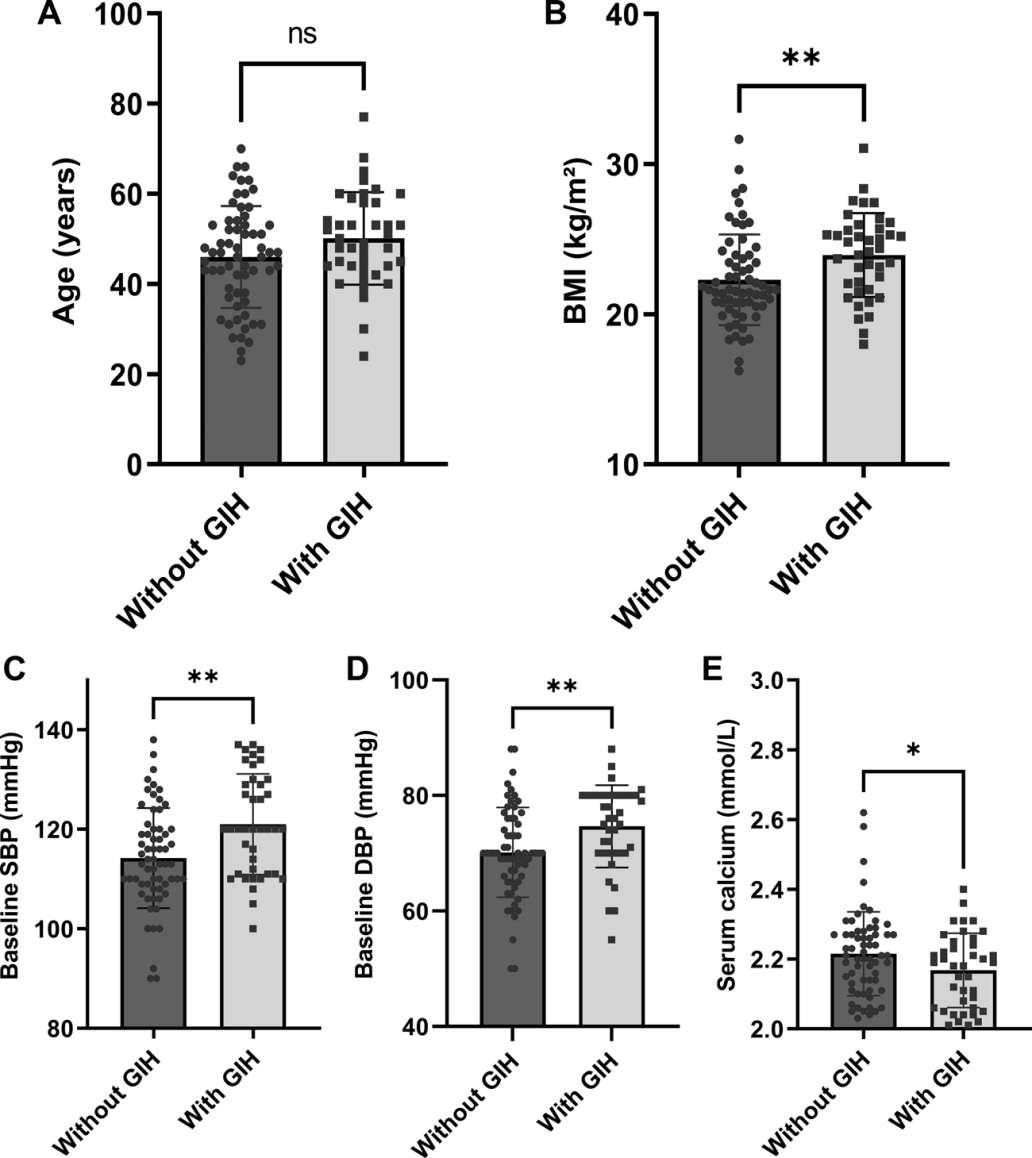
Comparison of baseline parameters between patients with and without glucocorticoid-induced hypertension (n = 103): **(A)** age, **(B)** BMI, **(C)** SBP, **(D)** DBP, and **(E)** serum calcium levels. Error bars depict mean ± standard deviation values. Individual data points are overlaid to demonstrate value distribution within each group. *p < 0.05; **p < 0.01. BMI, body mass index; SBP, systolic blood pressure; DBP, diastolic blood pressure; GIH, glucocorticoid-induced hypertension.

Factors possibly associated with glucocorticoid-induced hypertension were measured by univariate logistic regression analysis, and the results are presented in **Table 1**. Serum calcium was negatively associated with glucocorticoid-induced hypertension (p = 0.046). For each 0.1 mmol/L increase in serum calcium, the risk of glucocorticoid-induced hypertension was reduced by 32% (OR = 0.68, 95% CI = 0.46–0.99). Furthermore, glucocorticoid-induced hypertension was also significantly correlated with BMI (p = 0.009), baseline SBP (p = 0.002), baseline DBP (p = 0.006), and possibly age (p = 0.067). The data distributions of the patients with and without glucocorticoid-induced hypertension are shown in **Figure 2**.

### 3.3 Multivariate logistic regression analysis between serum calcium and glucocorticoid-induced hypertension

To examine the independent connection between serum calcium and glucocorticoid-induced hypertension, variables with p values less than 0.1 were added to multivariate logistic regression analysis along with serum calcium; thus, after adjusting for age, BMI, SBP, and DBP, a negative correlation was found between them in the basic model (p = 0.028). For each 0.1 mmol/L increase in serum calcium, the OR (95% CI) was 0.61 (0.39, 0.95). P values and ORs were not strongly affected when other possible confounders, such as the serum albumin concentration and glucocorticoid dose, were accounted for independently on the basis of the basic model (**Table 2**). In other words, the relationship between serum calcium and glucocorticoid-induced hypertension was stable.

**Table 2:** Associations of serum calcium with glucocorticoid-induced hypertension in the complete cohort (n = 103).

	Serum calcium For each 0.1 mmol/L increase	
	OR (95% CI)	P Value
Not adjusted	0.68 (0.46, 0.99)	0.046
Basic model*	0.61 (0.39, 0.95)	0.028
Basic model plus sex	0.63 (0.40, 0.98)	0.042
Basic model plus serum albumin	0.60 (0.37, 0.98)	0.041
Basic model plus glucocorticoid dose	0.61 (0.39, 0.95)	0.028
Basic model plus family history of hypertension	0.59 (0.37, 0.93)	0.023
Basic model plus smoking history	0.60 (0.38, 0.96)	0.031
Basic model plus drinking history	0.60 (0.38, 0.95)	0.028
Basic model plus thyroid function	0.61 (0.39, 0.95)	0.028
Basic model plus FPG	0.57 (0.36, 0.91)	0.018
Basic model plus TG	0.59 (0.38, 0.94)	0.024
Basic model plus TC	0.63 (0.40, 0.99)	0.043
Basic model plus LDL-C	0.62 (0.40, 0.98)	0.040
Basic model plus SCR	0.60 (0.38, 0.95)	0.028
Basic model plus calcium supplements	0.61 (0.39, 0.95)	0.029
Basic model plus vitamin D supplements	0.61 (0.39, 0.95)	0.030

*Adjusted for age, BMI, SBP, and DBP.

BMI, body mass index; SBP, systolic blood pressure; DBP, diastolic blood pressure; FPG, fasting plasma glucose; TG, triglyceride; TC, total cholesterol; LDL-C, low-density lipoprotein cholesterol; SCR, serum creatinine.

**Figure 3:**
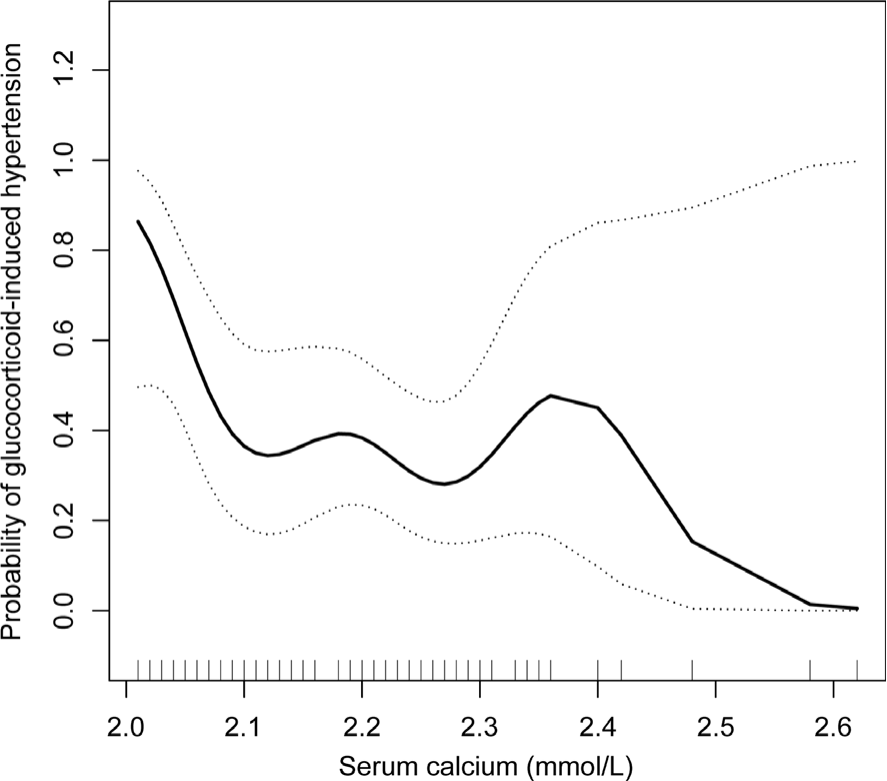
Relationships between the serum calcium concentration and glucocorticoid-induced hypertension after adjusting for age, BMI, SBP, and DBP in the complete cohort (n = 103). A nonlinear relationship and a saturation effect were observed, and the inflection point was 2.10 mmol/L. BMI, body mass index; SBP, systolic blood pressure; DBP, diastolic blood pressure.

A nonlinear association between the serum calcium concentration and glucocorticoid-induced hypertension after adjusting for age, BMI, SBP, and DBP was further shown in the smooth curve (**Figure 3**). The inflection point was 2.10 mmol/L (**Table 3**). On the left side of the inflection point, the risk of glucocorticoid-induced hypertension increased as the serum calcium level decreased (p = 0.028); however, on the right side of the inflection point, the two parameters were not related (p = 0.737).

**Table 3:** Threshold effect analysis of serum calcium on glucocorticoid-induced hypertension via piecewise linear regression in the complete cohort (n = 103).

Inflection point of serum calcium	OR (95% CI) *	P Value
< 2.10	0.058 (0.005, 0.733)	0.028
≥2.10	1.131 (0.552, 2.317)	0.737

*For each 0.1 mmol/L increase in serum calcium and adjusted for age, BMI, SBP, and DBP.

BMI, body mass index; SBP, systolic blood pressure; DBP, diastolic blood pressure.

### 3.4 Association of hypocalcemia with glucocorticoid-induced hypertension

On the basis of the curve inflection point (2.10 mmol/L), serum calcium was modeled as a categorical variable, and patients with a serum calcium concentration less than 2.10 mmol/L were classified as having hypocalcemia. According to the unadjusted model, there was a positive correlation between hypocalcemia and glucocorticoid-induced hypertension; however, the p value was slightly greater than 0.05 (p = 0.052) (**Table 4**). In the basic model adjusted for age, BMI, SBP, and DBP, this correlation was significant (p = 0.031), and the OR (95% CI) was 3.26 (1.11, 9.53). Consistent with earlier results concerning serum calcium mentioned above, the link between hypocalcemia and glucocorticoid-induced hypertension was largely unaffected by additional adjustment for other variables, with p values that were consistently < 0.05.

**Table 4:** Association of hypocalcemia with glucocorticoid-induced hypertension in the complete cohort (n = 103).

	Hypocalcemia	
	OR (95% CI)	P Value
Not adjusted	2.55 (0.99, 6.57)	0.052
Basic model*	3.26 (1.11, 9.53)	0.031
Basic model plus sex	3.02 (1.02, 8.91)	0.045
Basic model plus serum albumin	3.07 (1.01, 9.34)	0.048
Basic model plus glucocorticoid dose	3.40 (1.15, 10.03)	0.026
Basic model plus family history of hypertension	3.37 (1.14, 10.01)	0.029
Basic model plus smoking history	3.24 (1.09, 9.65)	0.034
Basic model plus drinking history	3.28 (1.11, 9.70)	0.032
Basic model plus thyroid function	3.30 (1.12, 9.71)	0.030
Basic model plus FPG	3.66 (1.21, 11.06)	0.022
Basic model plus TG	3.36 (1.14, 9.94)	0.028
Basic model plus TC	3.01 (1.01, 8.98)	0.048
Basic model plus LDL-C	2.99 (1.00, 8.95)	0.049
Basic model plus SCR	3.30 (1.12, 9.69)	0.030
Basic model plus calcium supplements	3.17 (1.08, 9.32)	0.036
Basic model plus vitamin D supplements	3.41 (1.15, 10.06)	0.026

*Adjusted for age, BMI, SBP, and DBP.

BMI, body mass index; SBP, systolic blood pressure; DBP, diastolic blood pressure; FPG, fasting plasma glucose; TG, triglyceride; TC, total cholesterol; LDL-C, low-density lipoprotein cholesterol; SCR, serum creatinine.

### 3.5 Association of hypocalcemia with glucocorticoid-induced BP fluctuations

**Figure 4:**
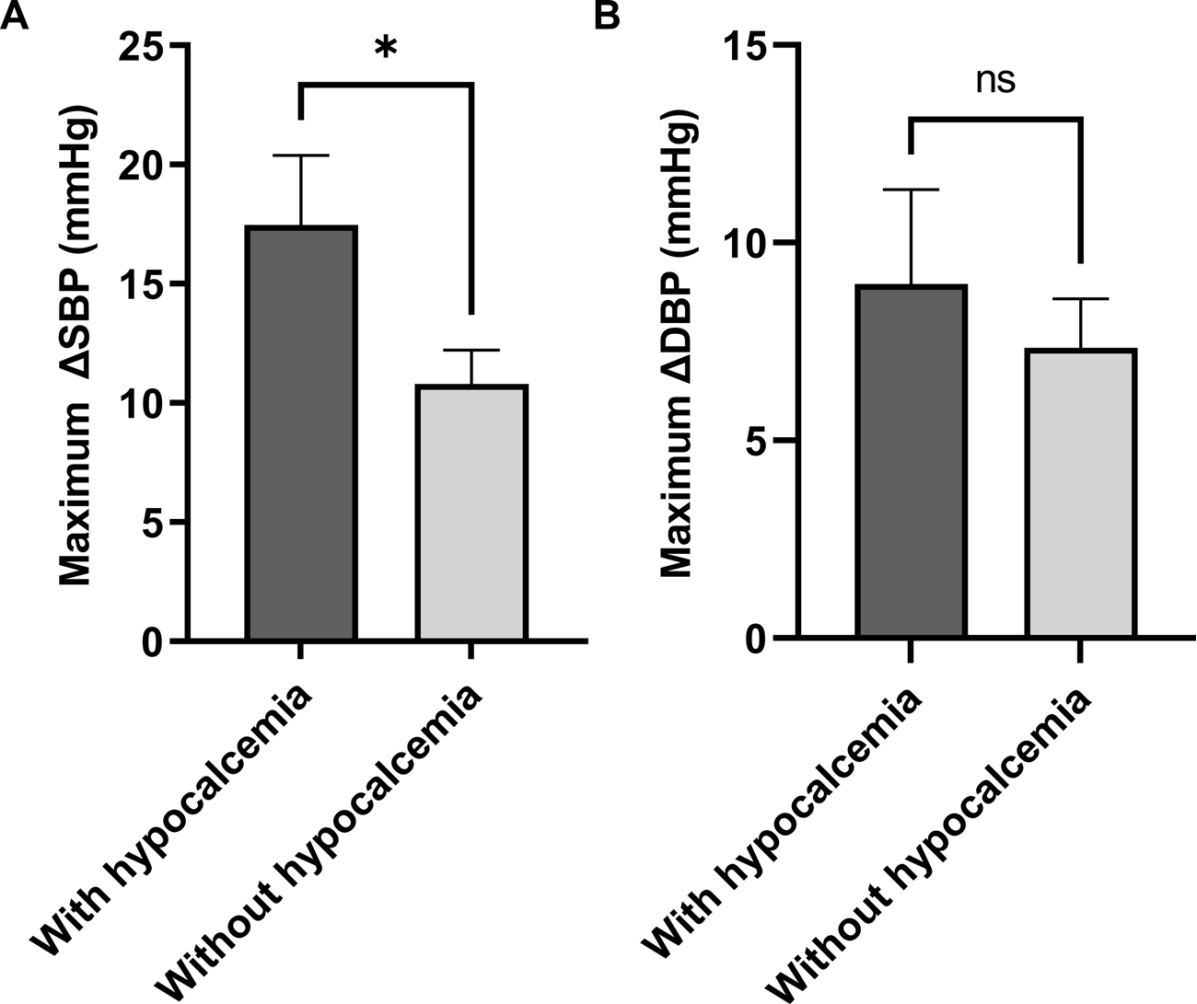
The relationship between hypocalcemia and glucocorticoid-induced BP fluctuations in the imputed cohort (n = 103). *p < 0.05. ΔSBP, systolic blood pressure difference; ΔDBP, diastolic blood pressure difference.

To evaluate whether hypocalcemia affects the range of BP fluctuations, patients were categorized into two groups on the basis of the inflection point of serum calcium (with hypocalcemia: < 2.1 mmol/L, without hypocalcemia: ≥2.1 mmol/L). The maximum SBP and DBP differences (ΔSBP and ΔDBP) were compared. Patients with hypocalcemia presented a significantly greater maximum ΔSBP than patients without hypocalcemia (p < 0.05). The maximum ΔDBP was also higher in patients with hypocalcemia, however, no significant difference was observed between the two groups (p > 0.05) (**Figure 4**).

### 3.6 Sensitivity analyses

All analyses were repeated in the imputed cohort (n = 135), and the results are shown in the **Supplementary File**. The relationship between serum calcium and glucocorticoid-induced hypertension was not significant (p = 0.121) according to the univariate logistic regression analysis (**Supplementary Table 1**). However, after adjusting for possible confounders, a significant negative correlation was found between them, and the p values were all less than 0.05 (**Supplementary Table 2**). The other results of the imputed cohort are similar to those of the complete cohort presented above (**Supplementary File**).

## 4 Discussion

In this retrospective cohort study, the percentage of TAO patients treated with IVMP who experienced glucocorticoid-induced hypertension was high (38.8%). Baseline SBP, DBP, and BMI were significantly associated with glucocorticoid-induced hypertension according to the univariate analyses. BMI is widely recognized as a risk factor for hypertension. The correlation between higher BMI and the risk of glucocorticoid-induced hypertension may be due to the increased levels of aldosterone and vasoconstrictor mediators, such as angiotensin II and endothelin-1, in obese patients (29, 30). Consistent with a study of lenvatinib-induced hypertension in patients with thyroid cancer, high baseline blood pressure is a risk factor for developing hypertension. This may be due to subclinical vascular dysfunction amplifying glucocorticoid-induced vascular reactivity (31).

Serum calcium was negatively and nonlinearly correlated with glucocorticoid-induced hypertension. After the conversion of serum calcium to a categorical variable, there was a strong positive association between glucocorticoid-induced hypertension and hypocalcemia. The maximum ΔSBP of patients with hypocalcemia was significantly greater. Therefore, we speculate that hypocalcemia at baseline may be a risk factor for glucocorticoid-induced hypertension.

Ionized calcium, the active form of serum calcium, makes up approximately half of total serum calcium, whereas bound calcium primarily binds to albumin (32). Therefore, the serum albumin level has an effect on the level of active calcium. However, the negative correlation between serum calcium and glucocorticoid-induced hypertension was unaffected after serum albumin was considered a confounder for adjustment. The majority of patients received vitamin D and calcium supplements to either prevent or treat osteoporosis during treatment. However, in our study, the presence or absence of these supplements during treatment was not associated with the risk of developing glucocorticoid-induced hypertension. These findings indicate that calcium supplementation may be required before beginning IVMP therapy to avoid glucocorticoid-induced hypertension.

A study among the elderly population in Japan supports our results. Their study revealed that the serum calcium level in patients with glucocorticoid-induced hypertension was significantly lower than that in patients without glucocorticoid-induced hypertension (p < 0.05). In addition, serum calcium was negatively correlated with the increased range of mean BP (r = -0.29) (33). Although this study has several limitations, including the lack of controlling for possible confounders and the small sample size of only 35 patients, it is the first to report that low serum calcium levels are associated with glucocorticoid-induced hypertension in elderly individuals.

The association between hypocalcemia and hypertension may be driven by increased vascular resistance and blood volume. Hypocalcemia causes parathyroid hormone (PTH) release and calcitriol production, which raise intracellular calcium ([Ca^2+^]_i_) in vascular smooth muscle cells (VSMC) by activating L-type calcium channels.

As [Ca^2+^]_i_ rises, vasoconstriction occurs, which raises peripheral vascular resistance and blood pressure. In addition, hypocalcemia leads to the activation of the renin-angiotensin-aldosterone system directly or indirectly by increasing PTH production. VSMC [Ca^2+^]_i_ is further amplified by angiotensin II via the phospholipase C (PLC)/inositol trisphosphate (IP₃) pathway, and blood volume is increased by aldosterone-induced sodium and water retention (34). Baksi et al. reported that angiotensin II type 1 receptor (AT-1R) on adrenocortical cells in rats increases, and AT-1R on smooth muscle cells decreases after a low-calcium diet, leading to the increase of aldosterone synthesis and blood pressure (35). Moreover, a high-calcium diet increases urinary sodium excretion and prevents hypertension (36).

However, the notion that hypertension induced by synthetic glucocorticoids is caused by sodium and water retention has been challenged by numerous studies. Synthetic glucocorticoids are far less capable of preventing the excretion of sodium and water than endogenous cortisol. In previous studies, synthetic glucocorticoids increased BP, but urinary sodium excretion did not decrease (37, 38). In addition, spironolactone was not shown to control the increase in BP caused by synthetic glucocorticoids, although it blocks sodium and water retention (39).

According to previous studies, the imbalance between the contraction and relaxation of vascular smooth muscles, oxidative stress, and metabolic syndrome are related to hypertension caused by glucocorticoids (40, 41). The synthesis of vasoconstrictors such as endothelin, catecholamine, and angiotensin or the response of vascular smooth muscle to these vasoconstrictors are increased by glucocorticoids (42–45). Moreover, the levels of vasodilators such as nitric oxide and prostaglandins are also decreased by glucocorticoids (38, 46–48). In addition, the activation of the renin–angiotensin system is involved. Angiotensinogen, angiotensin converting enzyme, and angiotensin II, as well as the expression of AT-1R on vascular smooth muscle cells, are increased in response to treatment with glucocorticoids (45, 49–52).

Synthetic (exogenous) glucocorticoids possibly mainly increase the contraction of vascular smooth muscle, whereas hypocalcemia leads to an increase in blood volume and exacerbates the occurrence of hypertension after glucocorticoid therapy, according to the above literature.

However, this study has the following limitations. The serum calcium concentrations of the patients in our study were mainly between 2.0 and 2.4 mmol/L. This makes it impossible to observe the association when the serum calcium concentration is greater than 2.4 mmol/L. Only TAO patients treated with IVMP were included in this cohort, and 118 patients were excluded because of missing BP or serum calcium levels. The final cohort was somewhat limited in our study due to patients with moderate-to-severe TAO requiring IVMP therapy needing strict inclusion and exclusion, and patients with preexisting hypertension or abnormal baseline BP were excluded (n = 103).

## 5 Conclusions

In general, glucocorticoid-induced hypertension is very common in TAO patients, and lower serum calcium at baseline significantly increases the risk of the occurrence of glucocorticoid-induced hypertension. For patients with pretreatment hypocalcemia, we recommend close and possible home monitoring of BP during and after IVMP. More research is needed to confirm whether calcium supplementation before glucocorticoid therapy reduces the risk of glucocorticoid-induced hypertension.

## Data Availability

The raw data supporting the conclusions of this article will be made available by the authors, without undue reservation.
