# Supplementary material for: Negative association between serum calcium and glucocorticoid-induced hypertension in thyroid-associated ophthalmopathy patients treated with methylprednisolone
Source: Front Endocrinol (Lausanne). 2025 Apr 11;16:1548953. doi: 10.3389/fendo.2025.1548953 (PMC12021620; doi:10.3389/fendo.2025.1548953)
Supplement: Supplementary file 1 [file DataSheet1.docx]

Supplementary Table 1. Crude associations of glucocorticoid-induced hypertension with serum calcium and other potential risk factors in the imputed cohort (n = 135)

| Variable | Mean ± SD/n (%) | OR (95% CI) | P Value |
| --- | --- | --- | --- |
| Age, y | 47.9 ± 11.2 | 1.03 (0.99, 1.06) | 0.117 |
| BMI, kg/m² | 23.0 ± 3.0 | 1.17 (1.03, 1.33) | **0.015** |
| SBP, mmHg | 115.4 ± 10.8 | 1.06 (1.02, 1.10) | **0.002** |
| DBP, mmHg | 71.0 ± 7.9 | 1.08 (1.03, 1.14) | **0.002** |
| Serum calcium, mmol/L | 2.19 ± 0.12 | 0.77 (0.55, 1.07)* | 0.121 |
| Serum albumin, g/L | 41.0 ± 3.6 | 1.07 (0.97, 1.19) | 0.169 |
| Glucocorticoid dose, g | 5.1 ± 1.4 | 1.09 (0.85, 1.39) | 0.500 |
| FPG, mmol/L | 4.9 ± 1.3 | 1.00 (0.74, 1.35) | 0.987 |
| SCR, umol/L | 55.0 ± 11.9 | 1.02 (0.99, 1.05) | 0.138 |
| TG, mmol/L | 1.31 ± 0.75 | 1.32 (0.83, 2.10) | 0.244 |
| TC, mmol/L | 4.04 ± 0.80 | 0.80 (0.50, 1.27) | 0.343 |
| LDL-C, mmol/L | 2.38 ± 0.67 | 0.83 (0.48, 1.43) | 0.493 |
| CAS | 3.0 ± 1.2 | 0.98 (0.71, 1.35) | 0.883 |
| Female | 63 (46.7%) | 0.81 (0.39, 1.68) | 0.573 |
| Smoking history | 51 (37.8%) | 1.22 (0.58, 2.54) | 0.602 |
| Drinking history | 29 (21.5%) | 1.35 (0.57, 3.18) | 0.490 |
| Family history of hypertension | 20 (14.8%) | 0.87 (0.31, 2.44) | 0.789 |
| Thyroid function: |  |  |  |
| Euthyroidism | 5 (3.7%) | Reference |  |
| Hyperthyroidism | 125 (92.6%) | 0.73 (0.12, 4.55) | 0.738 |
| Hypothyroidism | 5 (3.7%) | 0.38 (0.02, 6.35) | 0.497 |
| Calcium supplements | 122 (90.4%) | 1.69 (0.44, 6.47) | 0.446 |
| Vitamin D supplements | 82 (60.7%) | 1.04 (0.50, 2.17) | 0.918 |

*For each 0.1 mmol/L increase in serum calcium.

Abbreviations: BMI, body mass index; SBP, systolic blood pressure; DBP, diastolic blood pressure; FPG, fasting plasma glucose; SCR, serum creatinine; TG, triglyceride; TC, total cholesterol; LDL-C, low-density lipoprotein cholesterol; CAS, clinical activity score.

Supplementary Table 2. Association of serum calcium with glucocorticoid-induced hypertension in the imputed cohort (n = 135)

|  | Serum calcium  For each 0.1 mmol/L increase | |
| --- | --- | --- |
|  | OR (95% CI) | P Value |
| Not adjusted | 0.77 (0.55, 1.07) | 0.121 |
| Basic model* | 0.65 (0.45, 0.95) | 0.028 |
| Basic model plus age | 0.67 (0.46, 0.98) | 0.041 |
| Basic model plus sex | 0.66 (0.46, 0.96) | 0.031 |
| Basic model plus serum albumin | 0.65 (0.45, 0.95) | 0.025 |
| Basic model plus glucocorticoid dose | 0.65 (0.44, 0.95) | 0.027 |
| Basic model plus family history of hypertension | 0.65 (0.44, 0.95) | 0.026 |
| Basic model plus smoking history | 0.64 (0.44, 0.95) | 0.026 |
| Basic model plus drinking history | 0.64 (0.44, 0.95) | 0.025 |
| Basic model plus thyroid function | 0.65 (0.44, 0.96) | 0.028 |
| Basic model plus FPG | 0.65 (0.44, 0.95) | 0.027 |
| Basic model plus TG | 0.65 (0.44, 0.95) | 0.027 |
| Basic model plus TC | 0.67 (0.45, 0.99) | 0.042 |
| Basic model plus LDL-C | 0.65 (0.44, 0.97) | 0.033 |
| Basic model plus SCR | 0.65 (0.45, 0.96) | 0.028 |
| Basic model plus calcium supplements | 0.65 (0.43, 0.99) | 0.043 |
| Basic model plus vitamin D supplements | 0.65 (0.43, 0.99) | 0.043 |

*Adjusted for BMI, SBP, and DBP.

Abbreviations: BMI, body mass index; SBP, systolic blood pressure; DBP, diastolic blood pressure; FPG, fasting plasma glucose; TG, triglyceride; TC, total cholesterol; LDL-C, low-density lipoprotein cholesterol; SCR, serum creatinine.


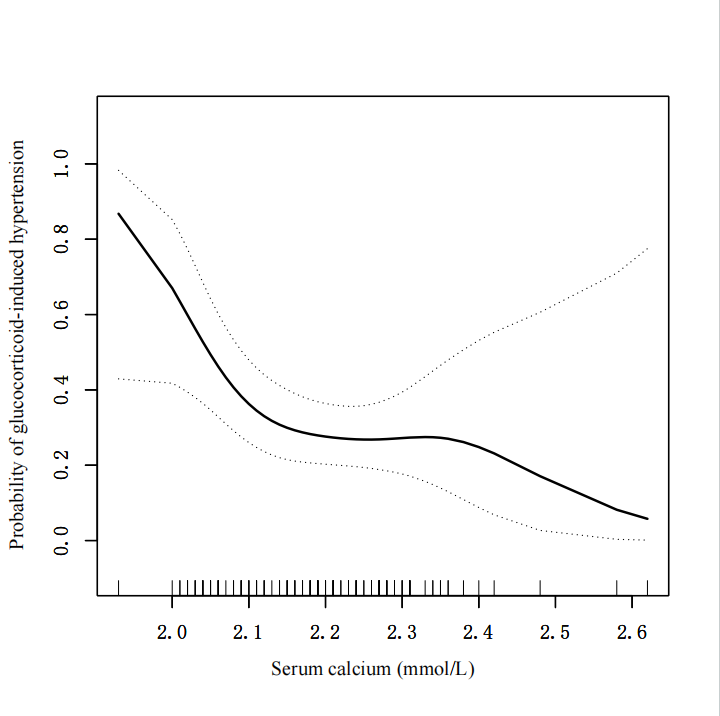


Supplementary Figure 1. Relationship between the serum calcium concentration and glucocorticoid-induced hypertension after adjusting for BMI, SBP, and DBP in the imputed cohort (n = 135). A nonlinear relationship and a saturation effect were observed, and the inflection point was 2.07 mmol/L.

Abbreviations: BMI, body mass index; SBP, systolic blood pressure; DBP, diastolic blood pressure.

Supplementary Table 3. Threshold effect analysis of serum glucocorticoid-induced hypertension via piecewise linear regression in the imputed cohort (n = 135)

| Inflection point of serum calcium | OR (95% CI) * | P Value |
| --- | --- | --- |
| < 2.07 | 0.053 (0.004, 0.630) | 0.020 |
| ≥2.07 | 0.861 (0.556, 1.333) | 0.501 |

*For each 0.1 mmol/L increase in serum calcium and adjusted for BMI, SBP, and DBP.

Abbreviations: BMI, body mass index; SBP, systolic blood pressure; DBP, diastolic blood pressure.

Supplementary Table 4. Association of hypocalcemia with glucocorticoid-induced hypertension in the imputed cohort (n = 135)

|  | Hypocalcemia | |
| --- | --- | --- |
|  | OR (95% CI) | P Value |
| Not adjusted | 2.47 (1.00, 6.07) | 0.049 |
| Basic model* | 3.89 (1.40, 10.79) | 0.009 |
| Basic model plus age | 3.76 (1.35, 10.48) | 0.011 |
| Basic model plus sex | 3.78 (1.36, 10.51) | 0.011 |
| Basic model plus serum albumin | 4.03 (1.43, 11.38) | 0.008 |
| Basic model plus glucocorticoid dose | 4.23 (1.50, 11.94) | 0.007 |
| Basic model plus family history of hypertension | 3.91 (1.41, 10.87) | 0.009 |
| Basic model plus smoking history | 3.93 (1.41, 10.95) | 0.009 |
| Basic model plus drinking history | 3.94 (1.41, 11.00) | 0.009 |
| Basic model plus thyroid function | 3.93 (1.41, 10.97) | 0.009 |
| Basic model plus FPG | 3.90 (1.41, 10.81) | 0.009 |
| Basic model plus TG | 3.92 (1.41, 10.88) | 0.009 |
| Basic model plus TC | 3.63 (1.29, 10.23) | 0.015 |
| Basic model plus LDL-C | 3.65 (1.29, 10.31) | 0.015 |
| Basic model plus SCR | 4.04 (1.44, 11.30) | 0.008 |
| Basic model plus calcium supplements | 3.36 (1.15, 9.80) | 0.026 |
| Basic model plus vitamin D supplements | 3.52 (1.20, 10.28) | 0.022 |

*Adjusted for BMI, SBP, and DBP.

Abbreviations: BMI, body mass index; SBP, systolic blood pressure; DBP, diastolic blood pressure; FPG, fasting plasma glucose; TG, triglyceride; TC, total cholesterol; LDL-C, low-density lipoprotein cholesterol; SCR, serum creatinine.


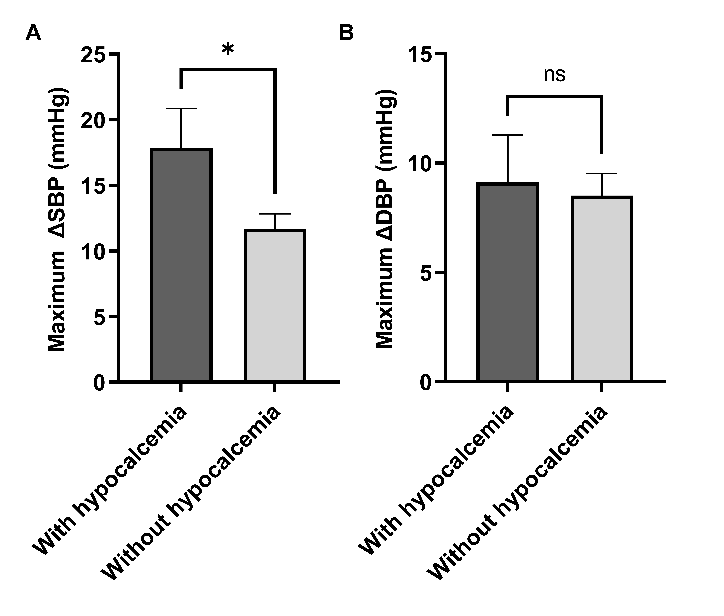


Supplementary Figure 3. The relationship between hypocalcemia and glucocorticoid-induced BP fluctuations in the imputed cohort (n = 135). *p < 0.05.

Abbreviations: ΔSBP, systolic blood pressure difference; ΔDBP, diastolic blood pressure difference.

Supplementary Table 4. Association of serum phosphorus with glucocorticoid-induced hypertension (n = 59)

| Variable | Mean ± SD/n (%) | OR (95% CI) | P Value |
| --- | --- | --- | --- |
| Serum phosphorus, mmol/L | 1.14 ± 0.19 | 1.39 (0.08, 24.66) | 0.822 |
